# Germ line transformation and in vivo labeling of nuclei in Diptera: report on *Megaselia abdita* (Phoridae) and *Chironomus riparius* (Chironomidae)

**DOI:** 10.1007/s00427-015-0504-5

**Published:** 2015-06-05

**Authors:** Francesca Caroti, Silvia Urbansky, Maike Wosch, Steffen Lemke

**Affiliations:** Centre for Organismal Studies, Universität Heidelberg, Im Neuenheimer Feld 230, 69120 Heidelberg, Germany

**Keywords:** Germ line transformation, *Chironomus riparius*, *Megaselia abdita*, EvoDevo

## Abstract

To understand how and when developmental traits of the fruit fly *Drosophila melanogaster* originated during the course of insect evolution, similar traits are functionally studied in variably related satellite species. The experimental toolkit available for relevant fly models typically comprises gene expression and loss as well as gain-of-function analyses. Here, we extend the set of available molecular tools to *piggyBac*-based germ line transformation in two satellite fly models, *Megaselia abdita* and *Chironomus riparius*. As proof-of-concept application, we used a Gateway variant of the *piggyBac* transposon vector pBac{3xP3-eGFPafm} to generate a transgenic line that expresses His2Av-mCherry as fluorescent nuclear reporter ubiquitously in the gastrulating embryo of *M. abdita*. Our results open two phylogenetically important nodes of the insect order Diptera for advanced developmental evolutionary genetics.

**Electronic supplementary material:**

The online version of this article (doi:10.1007/s00427-015-0504-5) contains supplementary material, which is available to authorized users.

## Introduction

**Fig. 1:**
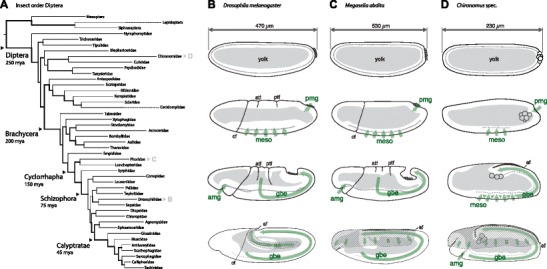
The insect order Diptera as evolutionary framework for early embryonic morphogenesis. **a** Dipteran phylogeny, with approximate age of last common ancestor given for major taxonomic groups (Wiegmann et al. 2011). **b–d** Schematic drawings and descriptions of successive stages of embryonic morphogenesis during gastrulation of *Drosophila melanogaster* (**b**), *Megaselia abdita* (**c**), and *Chironomus spec* (**d**), modified from and based on (Campos-Ortega and Hartenstein 1997; Lye and Sanson 2011; Rafiqi et al. 2008; Wotton et al. 2014; Ritter 1890) and own observations of fixed and live embryos. **b** Just prior to gastrulation, embryonic development in *D. melanogaster* is characterized by cellularization of the initially syncytial blastoderm. Germ cells have formed and are characterized by their round shape and position at the posterior pole of the cellularizing blastoderm. Gastrulation starts with the involution of the presumptive mesoderm along the ventral midline of the embryo, which first forms a furrow and then an epithelial tube that eventually collapses. Concurrent with mesoderm involution, the cephalic furrow forms, the posterior pole plate tilts, and the germband elongates by convergent extension of the lateral ectoderm. During germband extension, dorsal folds form between cephalic furrow and the front of the germband, and pole cells as well as anterior and posterior midgut are internalized. Cells of the dorsal blastoderm flatten and stretch to give rise to an extraembryonic epithelium (amnioserosa), which covers the dorsal opening of the embryo until its closure after germband retraction. **c** Gastrulation in *M. abdita* is qualitatively similar to *D. melanogaster*. Major differences compared to *D. melanogaster* have been described for extraembryonic development, which, in *M. abdita*, originates from the dorsal blastoderm like in *D. melanogaster*. In contrast to *D. melanogaster*, however, the amnioserosal fold extends further in *M. abdita*, ruptures, and gives rise to a serosa that detaches from the embryo proper, crawls over, and eventually encloses it. **d** Gastrulation in Chironomus shares with *M. abdita* and *D. melanogaster* the overall domains of the blastoderm embryo that give rise to endoderm, mesoderm, and ectoderm and the extraembryonic epithelia; and, like in other flies, the germband of Chironomus elongates by convergent extension. In contrast to *M. abdita* and *D. melanogaster*, Chironomus additionally shares characteristics with embryonic development of non-dipteran insects: blastoderm cellularization leads to a much less-pronounced columnar epithelium; pole cells are internalized during cellularization, and prior to the onset of germband extension, Chironomus embryos lack a cephalic furrow, mesoderm invagination is neither characterized by a deep furrow nor a ventral tube, and prominent dorsal folds cannot be observed during germband extension. The dorsal blastoderm develops by extension of the amnioserosal fold into the extraembryonic serosa. Unlike in *M. abdita*, the serosa does not detach from the remaining embryo and fuses, like in *Tribolium castaneum*, on the ventral side of the embryo and thus forms a ventral amnion. *cf* cephalic furrow, *meso* mesoderm, *pmg* posterior midgut, *amg* anterior midgut, *atf* anterior transverse furrow, *ptf* posterior transverse furrow, *gbe* germband extension, *af* amniotic fold, *as* amnioserosa, *s* serosa; *gray area*: yolk; *dotted lines in top row*: ingrowing front of cell membranes; *green arrows*: global cell movements; *area with oblique lines*: extraembryonic tissue, amnioserosa in *D. melanogaster* and serosa in *M. abdita* and Chironomus. In all schematics, anterior is to the *left* and dorsal *up*

The insect order Diptera (true flies, Fig. 1a) aggregates several traits that have facilitated its use as framework to link the molecular evolution of genomes and genetic networks with phenotypic divergence and novelty across consecutive macroevolutionary timescales (Rafiqi et al. 2011). Diptera have been studied for over a century, they cover about 250 million years of insect radiation within a well-established phylogeny, and they contain *Drosophila melanogaster*, one of the best-studied model systems in developmental biology (Anderson 1966; Wiegmann et al. 2011). Common traits of early embryonic development in flies are the absence of a posterior growth zone and segmentation within the blastoderm embryo prior to gastrulation (long germ insects, Davis and Patel 2002). During fly gastrulation, the lateral ectoderm elongates by convergent extension (germband extension), midgut precursors involute at the anterior and posterior pole, the mesoderm internalizes on the ventral side, the lateral ectoderm gives rise to neuronal progenitors, and extraembryonic tissue forms from cells of the dorsal blastoderm (Fig. 1b–d, Anderson 1966). The molecular basis of fly segmentation and morphogenesis is best understood in *D. melanogaster* (Fig. 1b). In *D. melanogaster*, saturated genetic screens and the molecular reconstruction of developmental circuits that pre-pattern the blastoderm embryo have established a powerful genetic reference system (Jaeger et al. 2012), to which development of satellite fly species can be compared (Cicin-Sain et al. 2015; Lemke et al. 2010). Two such satellite fly models, which have been established for these purposes at informative positions within the dipteran phylogeny, are *Megaselia abdita* (Phoridae) and *Chironomus riparius* (Chironomidae) (Fig. 1a).

The scuttle fly *M. abdita* represents the basal branch of cyclorrhaphan flies; it shared the last common ancestor with *D. melanogaster* approximately 145 million years ago and has been introduced as comparative system to bridge major differences in embryonic development of mosquito-related flies (Culicomorpha) and *D. melanogaster* (Rafiqi et al. 2011). While overall similar to Drosophila (Fig. 1c), embryonic development in *M. abdita* retained ancestral features that have helped to understand the evolution of highly diverged traits of the Drosophila model, such as the transition from an ancestral bipartite extraembryonic development (formation of a separate amnion and serosa) toward the amnioserosa as a single extraembryonic epithelium in *D. melanogaster* (Rafiqi et al. 2012).

The midge *C. riparius* represents the nematoceran suborder, which constitutes the basal branch of this insect order and shared the last common ancestor with *D. melanogaster* approximately 250 million years ago (Wiegmann et al. 2011). Within the nematoceran suborder, Chironomidae are closely related and share common developmental traits with mosquitoes (Culicidae) (Anderson 1966) but are less laborious to maintain, easy to manipulate, accessible for in vivo imaging, and have a long history of being used in developmental studies (reviewed in Sander 2000).

For *M. abdita* and *C. riparius*, transcriptome and genome resources are either available or in the process of being established (Jimenez-Guri et al. 2013; Marinković et al. 2012; S. Lemke and U. Schmidt-Ott, unpublished). Embryos of *M. abdita* have been shown to be very suitable for functional studies (Rafiqi et al. 2012; Stauber et al. 2000); similar results have been obtained for embryos of *C. riparius* (Klomp et al. 2015). Both species, like many other non-drosophilid fly models used to study the evolution of development, lack protocols for stable germ line transformation. In *D. melanogaster*, germ line transformation has been used for in vivo functional dissection of *cis*-regulatory DNA, for precisely targeted gene expression and gene knockdown during later stages of embryogenesis, or for in vivo cell tracking and lineage analyses (St Johnston 2013; Rebollo et al. 2014). Germ line transformation in other insects has been achieved previously by using transposon elements such as *piggyBac*, *Hermes*, *Minos*, and *mariner* (Horn et al. 2002; O’Brochta and Atkinson 1996). Here, we report successful transgenesis in *M. abdita* and *C. riparius* based on the *piggyBac* transposon system. As proof of concept and tool for future cell tracking and lineage analyses, we generated a His2Av-mCherry fusion construct in *M. abdita*, which was expressed ubiquitously in the early gastrulating embryo.

## Material and methods

Details on fly culture maintenance, cloning procedures, germ line transformation, and the molecular analyses of transgenic lines are provided in Supplementary Methods.

### Fly cultures

*M. abdita* Schmitz (Sander strain) was obtained from Johannes Jäger (CRG, Barcelona, Spain); the culture descends from the *M. abdita* strain maintained in the Schmidt-Ott laboratory and was maintained as described (Rafiqi et al. 2011). *C. riparius* Meigen was obtained from Urs Schmidt-Ott (The University of Chicago, Chicago, USA), who obtained the culture from Gerald K. Bergtrom (University of Wisconsin, Milwaukee, WI) in 2004 (Klomp et al. 2015). The culture was maintained at 25 °C to 28 °C and a constant 17/7-h day/night cycle. Embryos, larvae, and pupae were reared in food safe containers (Cambro) as aqueous cultures with constant aeration. Larvae were fed with a suspension of food paste prepared from sterilized milled parsley (Tro-Kost) and active dry baker’s yeast (0.65 %, *w*/*w*); eclosed flies were collected regularly and transferred to a separate cage with a dish of water for the deposition of egg packages.

### Cloning procedures

To generate in vitro-transcribed messenger RNA (mRNA) of the *piggyBac* transposase, the coding sequence was amplified by PCR and cloned into pSP35; the resulting vector pSPiggyHelp was linearized by *Eco*RI, and capped mRNA was synthesized using mMessage mMachine SP6 transcription kit (Life Technologies). To allow for three-way Gateway assembly of DNA fragments into *piggyBac*, two *piggyBac* destination vectors were generated, pBacDestA{3xP3-eGFP} and pBacDestB{3xP3-eGFP}: PCR-amplified *ccdB* cassettes with flanking attR4 and attR3 sites were inserted into the *Asc*I and *Bgl*II sites of pBac{3xP3-eGFPafm} (Horn and Wimmer 2000) to generate pBacDestA{3xP3-eGFP} and pBacDestB{3xP3-eGFP}, respectively. The *piggyBac* vector pBacDest{His2Av-mCherry} was used to generate the transgenic nuclear reporter line His2Av/sqh::His2Av-mCherry; it was assembled by LR recombination of three Gateway entry vectors into pBacDestA{3xP3-eGFP}, i.e., 5′-pENTR-His2Av, which contained the *M. abdita His2Av* locus from position −585 to +834 (+1 being the beginning of the ORF) followed by a fragment encoding the 20 C-terminal amino acids of *D. melanogaster His2Av*, middle-pENTR-mCherry, which contained the *mCherry* CDS, and 3′-pENTR-sqh, which contained 1077 bp immediately downstream of the *M. abdita spaghetti squash* CDS.

### Germ line transformation

*M. abdita* embryos were injected at the posterior pole with pre-mixed pBac{3xP3-eGFP} plasmid and in vitro-synthesized mRNA encoding the *piggyBac* transposase at DNA/RNA concentrations of 100:300 ng/μl. *C. riparius* embryos were injected into the center with pre-mixed plasmid and mRNA at DNA/RNA concentrations of 500:300 ng/μl or 100:300 ng/μl. Screening for enhanced Green Fluorescent Protein (eGFP) expression either in the adult eye (*M. abdita*) or in the nervous system of late-stage larvae (*C. riparius*) was performed using fluorescent binoculars (Olympus MVX 10, light source: X-cite Series 120Q; Nikon AZ 100, light source: Nikon Intensilight C-HGFI). To maintain transgenic lines, individuals were constantly inbred and screened for the fluorescent reporter in each generation.

### Microscopy and image analysis

Expression of His2Av-mCherry in early developing *M. abdita* embryos was analyzed on a MuVi-SPIM. Briefly, embryos were obtained from 30- to 45-min depositions, dechorionated, washed, and mounted for imaging as described previously for *D. melanogaster* (Krzic et al. 2012). For the calculation of nuclei number, image stacks were segmented with iLastik (1.0) and the position of each nucleus was extracted with Matlab (R2013a). Nuclear density was measured manually by counting the number of nuclei within a defined area.

## Results and discussion

### *piggyBac*-mediated germ line transformation in *M. abdita*

To establish *piggyBac*-based germ line transgenesis in *M. abdita*, a standard 3xP3-eGFP *piggyBac* vector was used, which, in *D. melanogaster*, drives eGFP reporter expression in the adult eyes and the larval nervous system (Horn et al. 2000). *M. abdita* embryos were collected from 30-min depositions (25 °C), covered with halocarbon oil, and injected through the chorion. Vector and in vitro-synthesized mRNA encoding the *piggyBac* transposase were pre-mixed and injected at the posterior pole of *M. abdita* embryos, which was determined by its rounder, less pointy tip, its larger diameter, and a retraction of the embryo from the vitelline membrane prior to the formation of pole cells.

**Fig. 2:**
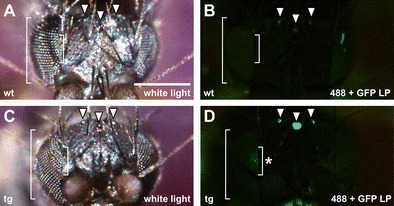
Expression of 3xP3-eGFP in transgenic *M. abdita*. **a**–**d** Heads of *M. abdita* wild type (*wt*; **a**, **b**) and transgenic fly (*tg*; **c**, **d**) shown with white light (**a**, **c**) and fluorescent illumination using a GFP long-pass filter set (488 + GFP LP; **b**, **d**). Transgenic animals showed fluorescence in ocelli (*arrowheads*) and ommatidia (*brackets*, *asterisk* in **d**) as reported for *D. melanogaster* (Horn et al. 2000). Presumably due to the dark pigmentation, fluorescence in the ommatidia is restricted to a small area of the ommatidia that are directly facing the microscope lens. *Scale bar* (in **a**) is 0.2 mm

Following injection, embryos were kept under oil in a moist chamber at 25 °C until hatching. After around 28 h, larvae started to hatch and were transferred to *M. abdita* culture vials; about 1 week after injection, the larvae pupated; 3 weeks after injection, the adult flies eclosed. Of 1100 injected embryos, 103 larvae hatched (103/1100 = 9.4 %), and 38 adult flies eclosed (G_0_, 38/103 = 36.9 %). Crosses among G_0_ flies were set up in small pools, and adults were screened after eclosure for eGFP expression in the eyes. Despite strong and dark pigmentation of the eyes, flies could be positively scored based on a specific green fluorescent signal in the compound eyes and ocelli (Fig. 2a–d); screening for eGFP expression in the larval nervous system was not possible due to autofluorescence of fish food in the gut. Based on green fluorescence in the eyes, transgenic animals were obtained from two independent G_0_ crosses, indicating a germ line transformation rate of 5.2 % (2/38). Green fluorescence in the eyes varied depending on the age of the flies, but throughout the lifetime of an adult it was strong enough to distinguish transgenic from wild type. To stably maintain a transgenic insertion, Drosophila genetics offer balancer chromosomes, which carry visible markers, suppress genetic recombination, and are homozygous lethal. In *M. abdita*, balancer chromosomes are not available. To maintain transgenic lines in *M. abdita*, flies were inbred and each generation was screened for green fluorescence in the eyes.

### Using germ line transformation to generate fluorescent nuclear in vivo reporter for *M. abdita*

Recent analyses in *D. melanogaster* and the flour beetle *Tribolium castaneum* have demonstrated how the dynamics of early embryonic development and gastrulation can be captured and quantitatively analyzed by employing ubiquitous fluorescent cell labeling in combination with in toto high-speed imaging (Krzic et al. 2012; Strobl and Stelzer 2014). In *D. melanogaster*, Histone2Av fused to a fluorescent protein is widely used to label nuclei as proxy for cell position with a high signal to noise ratio during all stages of the cell cycle (Krzic et al. 2012). To test whether *piggyBac*-based germ line transformation could be used to generate an equivalent tool in *M. abdita*, we first identified the *M. abdita* orthologue of the *His2Av* locus, including 0.5 kb of putative 5′ regulatory region in conserved synteny with *bällchen*. Poor genome assembly in the 3′ genomic region of *M. abdita His2Av* precluded cloning of its 3′ UTR and regulatory DNA that may account for ubiquitous gene expression. The missing sequence information was substituted with the 3′ UTR of *M. abdita spaghetti squash*, which, in *D. melanogaster*, encodes the ubiquitously expressed regulatory myosin light chain (Kiehart et al. 2000). The different DNA fragments were combined by three-way Gateway reaction into one of two newly generated *piggyBac* destination vectors (pBacDest{His2Av-mCherry}, Supplemental Fig. 1), and injection of pBacDest{His2Av-mCherry} for germ line transformation yielded 92 larvae (92/2100 injected embryos = 4.3 %), of which 36 adult flies eclosed (G_0_, 36/92 = 39.1 %). Crosses among G_0_ flies were set up in small pools as outlined above. In two independent crosses, transgenic offspring was identified based on green fluorescence in the eyes (2/36 = 5.5 %) and used to establish two independent His2Av/sqh::His2Av-mCherry lines through repeated inbreeding.

**Fig. 3:**
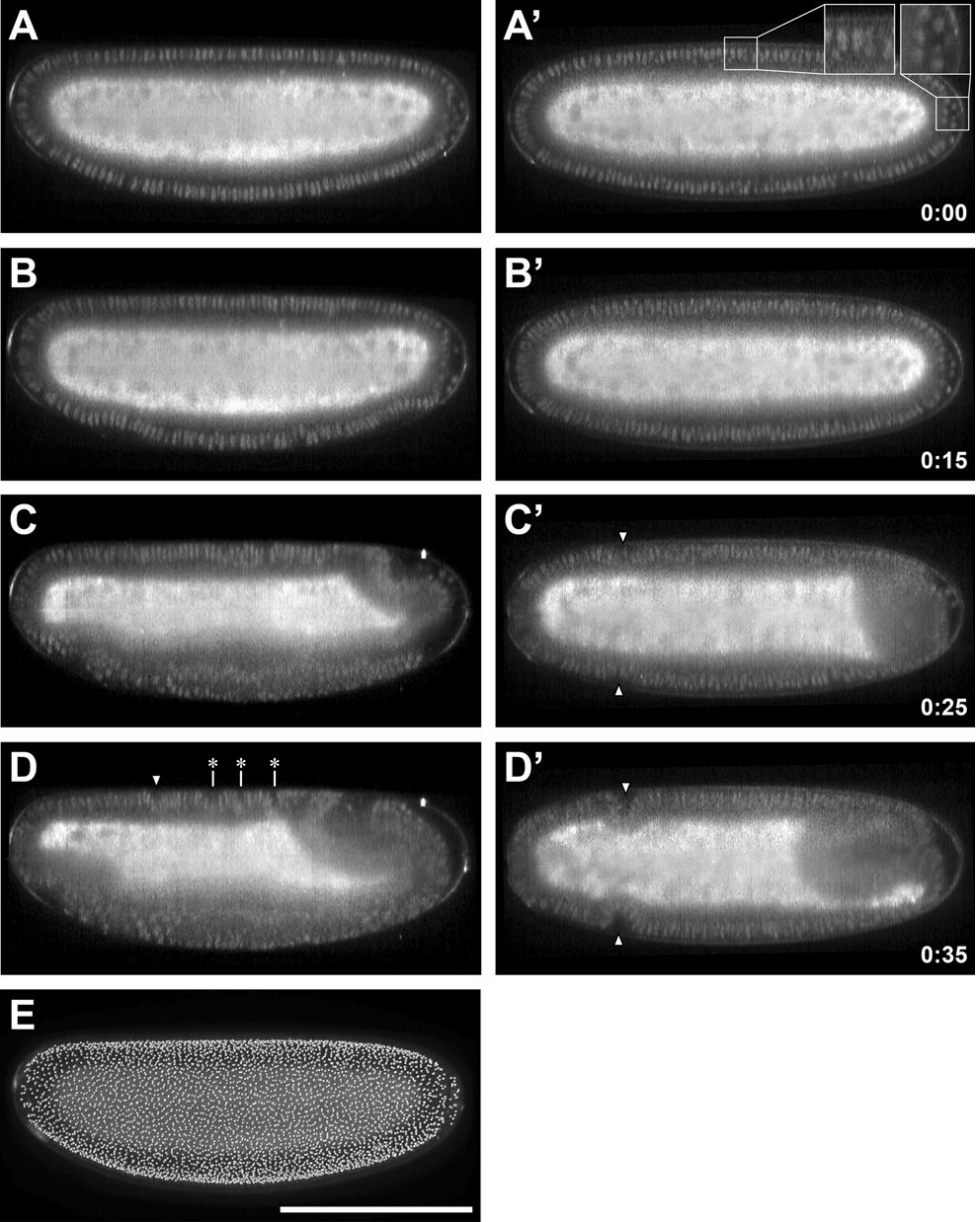
His2Av-mCherry expression in *M. abdita* embryos at late blastoderm stages and during the onset of gastrulation. **a–d’** Embryos are shown as mid-sagittal (*left column*) and transverse sections (*right column*) in late blastoderm stage (**a**, **a’**), at the onset of gastrulation (**b**, **b’**), briefly after the onset (**c**, **c’**), and during germband extension (**d**, **d’**). During late blastoderm stage, nuclei in the periphery were elongated and could be distinguished from the more spherical shape of the pole cells (*insets* in **a’**). Onset of cephalic furrow formation was observed after the onset of germband extension (*white triangles* in **c’**, **d**, **d’**); dorsal folds appeared along the dorsal midline during germband extension (*asterisk* in **d**). **e** Overlay of nuclear positions (*gray spheres*) extracted by automated image segmentation and raw image of embryo at late blastoderm stage. The embryos showed additional fluorescence in the yolk, which was significantly higher than in non-transgenic flies and suggested that not all of the His2Av-mCherry fusion protein was associated with chromatin. All embryos are shown with anterior to *left*; *scale bar* (in **e**) is 200 μm

To analyze how His2Av/sqh::His2Av-mCherry was expressed in *M. abdita*, we imaged the transgenic line in a late blastoderm embryo and during the onset of gastrulation using a multi-view light-sheet microscope (Krzic et al. 2012). The *M. abdita* His2Av/sqh::His2Av-mCherry line showed sufficient nuclear-associated fluorescence to allow for visual and computational segmentation of individual nuclei in the blastoderm. Compared with an analysis based on in vivo bright-field microscopy and fixed specimen (Wotton et al. 2014), we found overall development, the order of events, and the timing between individual developmental events accurately recapitulated (Fig. 3, Supplemental Movie 1): in late blastoderm stage, all nuclei in the periphery were elongated and could be distinguished by their shape from the more spherical shape of the pole cells (Fig. 3a, a’); onset of mesoderm internalization was characterized by basally descending nuclei along the ventral midline (Fig. 3b, b’) and was followed about 10 min later by the onset of germband extension, formation of the ventral furrow, and a basal nuclear shift in the cephalic furrow initiator cells (Fig. 3c, c’). During germband extension, a third dorsal fold could be discerned in addition to two previously described transverse folds along the dorsal midline between cephalic furrow and the amnioproctodeal invagination (Rafiqi et al. 2008) (Fig. 3d, d’). The signal to noise ratio of His2Av-mCherry-labeled nuclei during blastoderm stage was sufficient to apply automated image segmentation routines and extract nuclear positions for embryos in the late blastoderm stage. Just before the onset of gastrulation, the *M. abdita* embryo contained, in total, 4534 nuclei in the periphery, of which 23 nuclei were classified as pole cell nuclei based on their position and spherical appearance (Fig. 3e). This corresponds to a mean nuclear density of 1.8 nuclei per 100 μm^2^, which is very similar to the 2.0 nuclei per 100 μm^2^ that have been measured in fixed and DAPI-stained material (Wotton et al. 2014). The His2Av-mCherry fusion protein continued to be ubiquitously expressed during later stages of embryonic development, but the signal to noise ratio decreased, and it was not longer possible to segment nuclei.

Based on green fluorescence in the eyes, the two transgenic His2Av/sqh::His2Av-mCherry lines were maintained for over 30 generations. In contrast to expression of the eGFP reporter, however, the expression of the His2Av-mCherry reporter was not stably maintained. After approximately ten generations, we observed continuous decrease in fluorescence independently in both lines, and after approximately 20 generations, expression of His2Av-mCherry in nuclei had been eventually lost. We were able to detect a single bona fide *piggyBac* insertion in the last maintained transgenic line (Supplemental Fig. 2). His2Av-mCherry expression in this line could not be restored after repeated outcrosses against wild-type flies, suggesting that expression loss had not been due to homozygousing of the flies. Expression of His2Av-mCherry could have been lost due to selective silencing of the *His2Av*-*mCherry* transgene. Alternatively, our transgenic lines had initially carried multiple *piggyBac* insertions as previously reported for *Bombyx mori* (Tamura et al. 2000) and *Ceratitis capitata* (Handler et al. 1998), and loss of *piggyBac* insertion copies over time led to the decreasing levels of *His2Av*-*mCherry* transgene expression. To avoid a possible loss of *piggyBac* insertions after germ line transformation, future transgenic lines will need to be screened for eGFP reporter as well as marker gene expression and, from the F_1_ onward, repeatedly outcrossed against wild type. Inbreeding of individual lines will then start only after the separation of potentially multiple *piggyBac* insertions. To independently increase overall expression levels of the *His2Av*-*mCherry* transgene, future *piggyBac* insertions could be generated to contain a tandem duplication of the *His2Av*-*mCherry* fusion locus.

### *piggyBac*-mediated germ line transformation in *C. riparius*

To test whether the procedure for germ line transformation in *M. abdita* was, in principle, applicable to other species within the insect order Diptera, we focused on the midge *C. riparius* as representative for the nematoceran suborder. *C. riparius* embryos were collected from <1-h-old egg packages, which were gently bleached to disintegrate the gelatinous string enclosing the individual eggs. Embryos were aligned on a glass slide along a capillary, briefly dried, and covered with halocarbon oil. While preliminary mock injections had indicated that even young embryos (i.e., 15–60 min after deposition of the egg package) survive physical penetration of the vitelline membrane, the survival rate decreased tremendously when injected with transposon and transposase prior to pole cell formation. All attempts to generate germ line transformation were therefore carried out by injecting *C. riparius* embryos at the two-pole-cell stage, or slightly later, into the center of the embryo.

Following injection, embryos were kept on the slide and under oil in a moist chamber at 25 °C. After about 3.5 days (i.e., roughly 12 h before injected larvae would hatch), the slides with the embryos were placed in a petri dish and water was slowly added until most of the oil detached from the glass and started to float on the water surface. Surviving embryos typically stayed with their vitelline membrane attached to remnants of oil on the glass of the capillary or the slide, and hatched larvae were found usually at the oil/water interface of these droplets. Following hatching, the larvae were transferred with a small loop to a food safe container with pre-aerated water and parsley suspension (see “Material and methods” section). Of 720 injected embryos, 27 larvae hatched (27/720 = 3.8 %). Because wild-type single crosses set up in 50-ml Falcon tubes showed fertilization rates below 10 %, all G_0_ adults were instead crossed in one single pool. From this G_0_ cross, five egg packages were obtained, each of which was let develop in a separate tank.

**Fig. 4:**
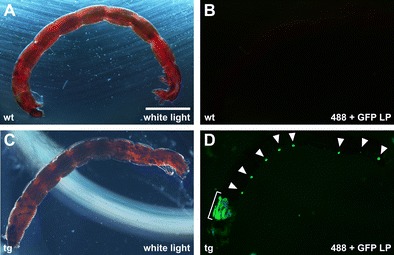
Expression of 3xP3-eGFP in *C. riparius*. **a**–**d** *C. riparius* wild type (*wt*; **a**, **b**) and transgenic larvae (*tg*; **c**, **d**) shown with white light (**a**, **c**) and fluorescent illumination using a GFP long-pass filter set (488 + GFP LP; **b**, **d**). Transgenic larvae showed fluorescence in the head (*bracket* in **d**) and individual segments (*arrowheads* in **d**) as reported for 3xP3-eGFP expression in the segmented nervous system of various dipteran larvae (Horn et al. 2000; Ito et al. 2002; Kim et al. 2004; Kokoza et al. 2001). *Scale bar* (in **a**) is 0.2 mm

*C. riparius* adult flies proved sensitive to CO_2_ and died after minimal exposure. However, as reported previously for *D. melanogaster*, *Aedes aegypti*, *Anopheles stephensi*, and *Anopheles gambiae* (Horn et al. 2000; Ito et al. 2002; Kim et al. 2004; Kokoza et al. 2001), we were able to observe fluorescence of eGFP in the nervous system of *C. riparius* larvae during the final instar stage, approximately 2 to 4 days prior to pupation. To identify putative transgenic animals, all F_1_ larvae were transferred during their last instar stage in batches of two to five individuals into wells of a 24-well plate and screened for eGFP expression in the central nervous system. From four out of five tanks, we obtained individual larvae showing a strong and specific green fluorescent signal in the larval brain and a segmental pattern throughout the abdomen (Fig. 4a–d). Since all transgenic animals were descendants of a single pooled G_0_ cross, we conservatively estimated germ line transformation at a rate of 3.7 % (1/27). Similar rates were obtained in two additional and independent experiments. The actual rate may be higher; not all G_0_ larvae eclosed and we obtained transgenic animals from more than two egg packages, which most likely stem from at least two independently transformed females present in the pooled G_0_ cross. Positive larvae were collected and transferred to separate tanks; pupation of these larvae followed after about 3 weeks; and by 4 weeks, latest, all adults had eclosed. F_1_ adults were intercrossed in a small plastic box, which contained the water tank for egg package deposition. To maintain the line, larvae were screened each generation for eGFP marker expression in the nervous system.

## Conclusions

Using the TTAA *piggyBac* element, we established protocols for successful germ line transformation in the two dipteran species *M. abdita* and *C. riparius*. With a net transformation rate of about 2–5 % per fertile G_0_ for *M. abdita* and *C. riparius*, our results are in the range of *piggyBac*-mediated germ line transformation events previously reported for other insects (Handler et al. 1998; Lorenzen et al. 2002; Pinkerton et al. 2000), suggesting that the rate of integration for *piggyBac* is relatively uniform throughout insects. In both our fly species analyzed, the overall survival rate of injected embryos was noticeably low and possibly affected by a high transposase activity provided through mRNA, suggesting that our protocol will benefit from additional fine-tuning of injection conditions.

With two new pBacDest vectors, we introduced Gateway variants of the widely used pBac{3xP3-eGFP} vector system, which extends *piggyBac*-based transgenesis in insects to ligation-free cloning, the fast and efficient assembly of multiple fragment constructs based on recombination, and the use of modular Gateway extensions such as Golden GATEway (Kirchmaier et al. 2013). As proof of concept, we have successfully tested this system in *M. abdita* by generating a transgenic line that expresses His2Av-mCherry as fluorescent nuclear reporter for in vivo time-lapse recordings. For the particular case of His2Av-mCherry as fluorescent nuclear reporter, the advent of targeted genome modification by CRISPR/Cas9 promises alternative paths to generate a similar reporter line by knock-in of a DNA fragment that encodes the fluorescent protein in frame with the endogenous coding sequences of *His2Av*. For other applications, e.g., ubiquitous expression of non-endogenous GAP43 as membrane marker, heterologous gene expression systems such as the GAL4/UAS system, or functional analyses of gene regulation via the fusion of putative *cis*-regulatory modules and reporter genes, classic germ line transformations remain a complementing and valuable tool and will significantly increase and extend the attraction of the insect order Diptera for in-depth developmental evolutionary studies.

## Electronic supplementary material

ESM 1 (PDF 2.61 mb)

ESM 2 (MP4 3262 kb)
