# Supplementary material for: Germ line transformation and in vivo labeling of nuclei in Diptera: report on Megaselia abdita (Phoridae) and Chironomus riparius (Chironomidae)
Source: Dev Genes Evol. 2015 Jun 5;225(3):179–86. doi: 10.1007/s00427-015-0504-5 (PMC4460289; doi:10.1007/s00427-015-0504-5)
Supplement: Supplementary file 1 — (PDF 2.61 mb) [file 427_2015_504_MOESM1_ESM.pdf]

Supplementary Figures

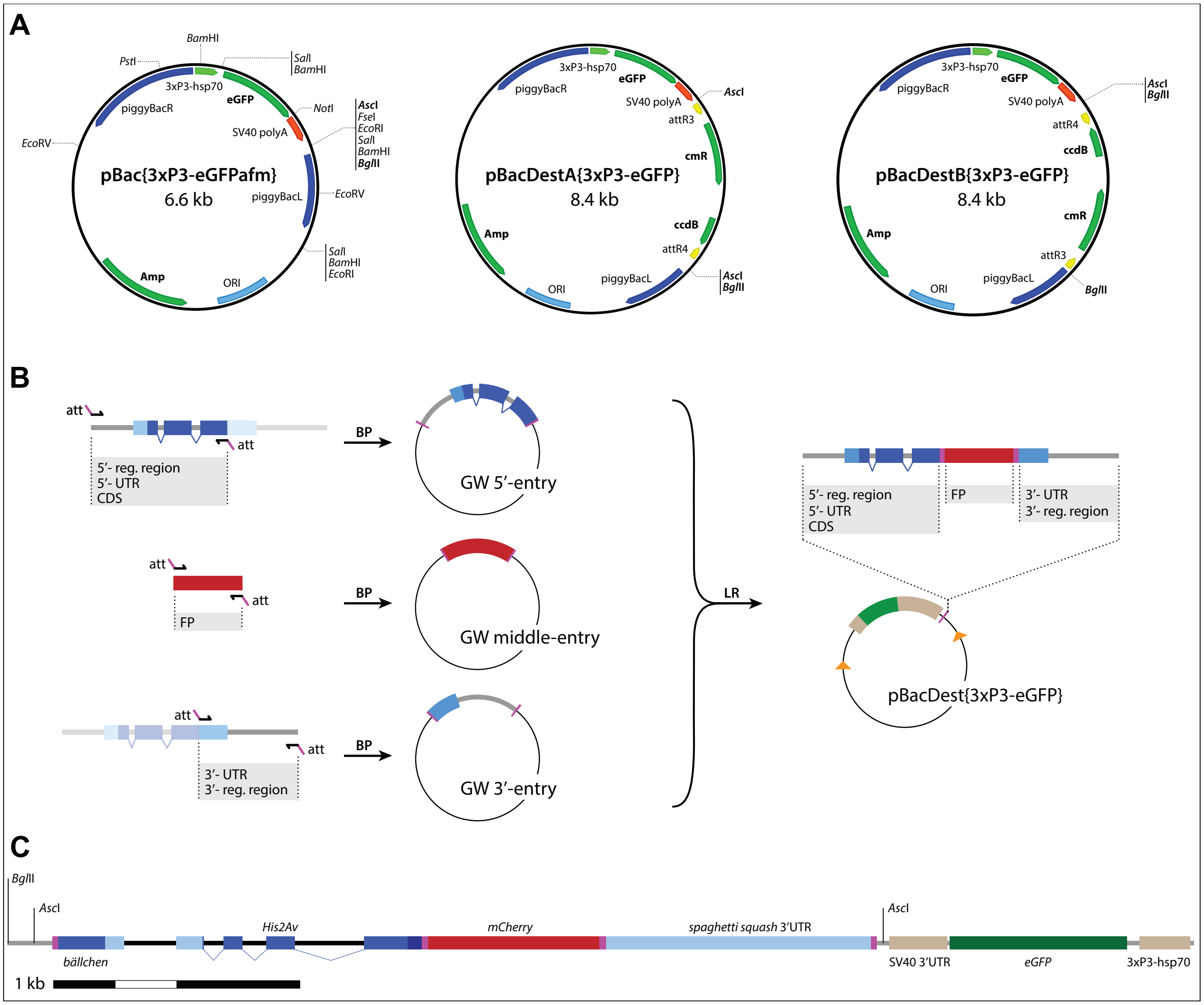

Supplemental Fig. 1 Gateway-based assembly of DNA fragments into *piggyBac* transposon system. (**A**) Schematic map of source plasmid pBac{3xP3-eGFPafm} (Horn and Wimmer, 2000) and derived destination vectors pBacDestA{3xP3-eGFP} and pBacDestB{3xP3-eGFP}. (**B**) Schematic overview illustrating how fragments containing (i) 5'-regulatory DNA, 5'-UTR and coding sequence, (ii) the coding sequence of a fluorescent protein, and (iii) the 3'-UTR and 3'-regulatory DNA are amplified by PCR using primers with att sites, cloned into the three respective Gateway entry vectors via BP-recombination (BP), and assembled into a three-way Gateway destination vector via LR-recombination (LR). (**C**) Depiction of pBacDest{His2Av-mCherry} with fully assembled insert between the piggyBac flanks of pBacDestA{3xP3-eGFP} according to scale: the fusion construct comprises 1.4 kb of the *M. abdita His2Av* locus, a C-terminus encoding fragment of *D. melanogaster His2Av*, coding sequence for mCherry fused in frame with the last exon of *His2Av*, and 1.1 kb of the *M. abdita spaghetti squash* locus, flanked by *Asc*I sites, as well as the transgenic reporter cassette consisting of the artificial eye promoter 3xP3-hsp70, the eGFP coding sequence and the SV40 3'UTR. Vector maps were generated with Geneious 8.0 and subsequently edited manually.

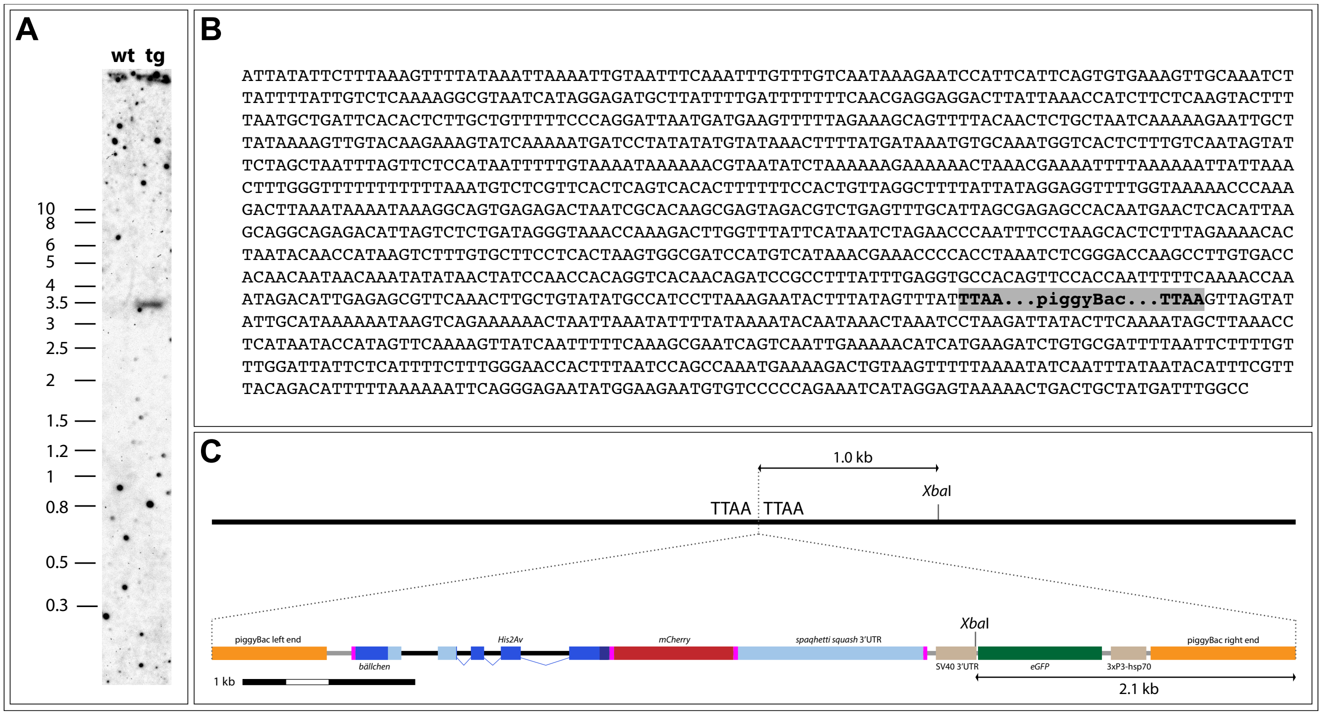

Supplemental Fig. 2 Characterization of genomic pBacDest{His2Av-mCherry} insertion in transgenic *M. abdita*. (**A**) The copy number of pBacDest{His2Av-mCherry} insertions was determined by southern blot hybridization with a probe against GFP; genomic DNA of wild type and transgenic flies had been digested with *Xba*I. The single *Xba*I band of around 3.4 kb in size suggests a single insertion in the genome of the maintained transgenic line. (**B**) Genomic sequence adjacent of the pBacDest{His2Av-mCherry} insertion was determined by inverse PCR, cloning, and sequencing of the obtained amplicons. All obtained sequences (16/16) mapped to a single insertion site that was identified on the basis of a preliminary genome assembly (Steffen Lemke, Thomas Sandmann, and Urs Schmidt-Ott, unpublished). Shown is the sequence flanking the *piggyBac* insertion in 5' to 3' orientation, insert and duplicated TTAA integration side are highlighted (bold font and gray shading). The duplicated TTAA integration site suggests that pBacDest{His2Av-mCherry} integrated as a bona fide *piggyBac* insertion into the genome of *M. abdita*. (**C**) Schematic overview of the site of pBacDest{His2Av-mCherry} integration into the *M. abdita* genome. Indicated are the two relevant *Xba*I sites, which result in a fragment comprising the eGFP coding sequence at a predicted size of about 3.1 kb.

Supplementary Methods

Fly cultures

Megaselia abdita wild type flies were kept on wet cotton and fish food (Tetra, TetraMin TE769939) in 250 ml plugged Drosophila bottles (Dutscher Scientific, 60 x 130 mm) at 25 °C, 65 % humidity, and on a 16/8-h day/night cycle. *M. abdita* transgenic flies were kept on wet cotton and fish food in 50 ml plugged Drosophila vials (Nerbe, 29 x 95 mm). To avoid mold formation, cotton in the small vials was moistened with 4-hydroxybenzoic acid methyl ester (Nipagin; stock solution: 10 % w/v in 70 % EtOH; diluted stock solution used in vials: 3 % v/v in water). Transgenic lines were maintained by constant inbreeding; each generation adults were screened for eGFP reporter expression.

Chironomus riparius embryos, larvae, and pupae were reared as aquatic cultures (tap water) in food safe containers with lids (Cambro Camwear® Pans). Wild type larvae were maintained in 8.9 liter containers (26CW, GN 1/4, 15 cm height) filled with about 5 liter of water, eclosed flies were collected regularly and transferred to a separate cage (30 x 35 x 40 cm) in which a dish filled with water was provided for egg package deposition. Egg packages of putative transgenic animals were individually raised in 2.2 liter containers (66CW, GN 1/6, 15 cm height) filled with about 2 liter, and injected larvae were raised in batches of up to thirty individuals in 0.85 liter containers (94CW, GN 1/9, 10 cm height) filled with about 0.6 liter of water. G_0 flies were crossed in a dedicated small deposition cage (15 x 20 x 15 cm). Transgenic lines were maintained by constant inbreeding; each generation larvae were screened for eGFP reporter expression.

Plasmid construction

pSPiggyHelp: To avoid low transcription and inefficient transposase synthesis due to variable promoter activity in a heterologous species context, transposase has been provided as mRNA, initially for *Minos* (Kapetanaki et al., 2002) and lately also for *piggyBac* (Bire et al., 2013). Following the same rationale, pSPiggyHelp had been generated as an expression vector for in vitro mRNA transcription of the *piggyBac* transposase. pSPiggyHelp was generated by amplifying the *piggyBac* transposase coding sequence (CDS) from phsp-pBac (Handler and Harrell, 1999) using primer pair 5'-CCAAACCATGG-GATGTTCTTTAGACGATG/5'-ATGAGTCGACTCAGAAACAACTTTGGCA and cloning of the *Nco*I/*Sal*I digested PCR product into pSP35 (Amaya et al., 1991). Plasmid DNA was prepared as QIAGEN midi prep followed by phenol/chloroform extraction, linearized by *Eco*RI, and capped mRNA of the transposase helper was in vitro-synthesized using mMessage mMachine SP6 transcription kit (Life Technologies).

pBacDestA{3xP3-eGFP} and *pBacDestB{3xP3-eGFP}*: Both three-way Gateway destination vectors were generated by insertion of *ccdB* cassettes with flanking attR4 and attR3 sites into the *Asc*I and *Bgl*II sites of pBac{3xP3-eGFPafm} (Horn and Wimmer, 2000), respectively. For pBacDestA{3xP3-eGFP}, the *ccdB* cassette was amplified from pDestTol2pA2 (Kwan et al., 2007) using primer pair 5'-AGTTGGCGCGCCGTGTCTGAAACACAGGCCAGAT/5'-ATGGCGCGCCGTAAAACGACGGC-CAGTGAATT, digested by *Asc*I, and cloned into the *Asc*I site of pBac{3xP3-eGFPafm}; for pBacDestB{3xP3-eGFP}, the *ccdB* cassette was amplified using primer pair 5'-ATGCTAGATCT-GTGTCTGAAACACAGGCCAGAT/5'-ATCGAAGATCTGTAAAACGACGGCCAGTGAATT, digested by *Bgl*II, and cloned into the *Bgl*II site of pBac{3xP3-eGFPafm}. The resulting vectors pBacDestA{3xP3-eGFP} and pBacDestB{3xP3-eGFP} constitute two independent Gateway destination vectors, of which pBacDestA{3xP3-eGFP} contains a single-cutter *Bgl*II, and pBacDestB{3xP3-eGFP} a single-cutter *Asc*I adjacent to the Gateway cassette (**Supplemental Fig. 1**). Through these complementing single-cutter sites a completely assembled three-fragment cassette of one pBacDest vector can be combined with the assembly of another pBacDest vector into one single *piggyBac* transposon vector by conventional cloning. The flanking *Asc*I and *Bgl*II sites in

pBacDestA{3xP3-eGFP} and pBacDestB{3xP3-eGFP} allow to introduce assembled three-fragment Gateway constructs into available *Hermes* and *mariner* based transposon vectors that contain single-cutter *Asc*I and/or *Bgl*II sites in their respective multiple cloning sites (Horn and Wimmer, 2000).

pBacDest{His2Av-mCherry}: The *piggyBac* vector pBacDest{His2Av-mCherry} containing the *M. abdita His2Av-mCherry-spaghetti squash* fusion construct was assembled by LR recombination of three Gateway entry vectors according to the Invitrogen user manual into pBacDestA{3xP3-eGFP}, i.e. 5'-pENTR-His2Av, middle-pENTR-mCherry, and 3'-pENTR-sqh. Entry vector 5'-pENTR-His2Av was obtained by BP reaction of the PCR amplified (iProof DNA Polymerase, BioRad) *M. abdita His2Av* locus into 5'-pENTR according to the Invitrogen user manual. The amplified *M. abdita His2Av* locus comprised position –585 to position +834 (+1 being the beginning of the ORF), plus a fragment encoding the 20 C-terminal amino acids of the *His2Av* CDS from *D. melanogaster* (+531 to +590, +1 being the beginning of the CDS in NM_079795), which was encoded by the reverse primer to complement for missing sequence information at the 3' end of the *M. abdita His2Av* CDS. The insert was amplified using primer pair 5'-GGGGACAACTTTGTATAGAAAAGTTGCTGCAGGATTGTT-TGCCTTGGTAG/5'-GGGGACT-GCTTTTTTGTACAAACTTGCGTAGGCCTGCGACAGAATGACGTTGCCCTTCCGCTGCGGA-TCCTGCACCGTTTCCTCCTTCTTTCCGATCAAAGATTTGTGGATATG and contained in-frame attB4 and attB1r sites for BP recombination; the template for this PCR reaction was obtained through cloning of a slightly larger fragment of the *M. abdita His2Av* locus, which was amplified by PCR (iProof DNA Polymerase, BioRad) from genomic DNA using primer pair 5'-GCAGGATTGTTTGCCTTGGTAG/5'-TCAAGGCCGAAAAGCACAAAAAC, A-tailed (Taq DNA polymerase F-100L, Finnzymes), and introduced into pCRII-TOPO (Invitrogen). Entry vector middle-pENTR-mCherry was obtained by BP reaction of the *mCherry* CDS into middle-pENTR. The CDS of *mCherry* was PCR amplified for BP recombination from plasmid pCaSpeR4-His2Av-mCherry (gift from Lars Hufnagel) by using primer pair 5'-GGGGACAAGTTTGTACAA-AAAAGCAGGCTTAATGGTGAGCAAGGGCGAGG/5'-GGGGACCACTTTGTACAAGAAAGC-TGGGTATTAGGCGCCGGTGGAGTGGC, and contained in-frame attB1 and attB2 sites. Entry vector 3'-pENTR-sqh was obtained by BP reaction of the PCR amplified *M. abdita spaghetti squash* 3'-UTR into 3'-pENTR by BP reaction. The amplified *M. abdita spaghetti squash* 3'-UTR contained 1,077bp immediately downstream of the *M. abdita spaghetti squash* CDS. The insert was PCR amplified for BP recombination by using primer pair 5'-GGGGACAGCTTTCTTGTACAAAGT-GGCTATGGAAAGAAACTGTCGA/5'-GGGGACAACTTTGTATAATAAAGTTGCAACCTCTTT-CGTCTCTTC, and contained attB2r and attB3 sites for BP recombination; the template for this PCR reaction was obtained through cloning of a slightly larger fragment of the *M. abdita spaghetti squash* locus, which was amplified by PCR from genomic DNA using primer pair 5'-GATGTACAGAGAAGCCCCGATTAAG/5'-CTAACAACCTCTTTCGTCTCTTCCAATCGC, A-tailed, and introduced into pCRII-TOPO.

Germ line transformation

M. abdita embryos were collected, lined up for injections, and covered with a 1+3 mixture of 27-halocarbon oil (Sigma H8773) and 700-halocarbon oil (Sigma H8898) as outlined previously (Rafiqi et al., 2011a; Rafiqi et al., 2011b). The pBac{3xP3-eGFP} plasmid and in vitro-synthesized mRNA encoding the *piggyBac* transposase were pre-mixed at DNA/RNA concentrations of 100:300 ng/µl and injected at the posterior pole of *M. abdita* embryos. Injection needles were pulled using a Flaming/Brown micropipette puller (Sutter Instruments P-97) and borosilicate glass capillaries (World Precision Instrument, 1.0 mm outer and 0.58 mm inner diameter). Needles were opened by beveling, backfilled using Eppendorf Microloader tips (0.5-20 µl), and injections were carried out using an Eppendorf FemtoJet express microinjector.

C. riparius embryos were collected from freshly deposited egg packages and briefly treated with 5% bleach to remove the gelatinous string enclosing the individual eggs (1- to 2- min). To remove the bleach, embryos were washed immediately and thoroughly with water. Embryos were lined up for injections, and covered sparingly with a 1+3 mixture of 27-halocarbon oil and 700-halocarbon oil essentially as described above for *M. abdita*. The pBac{3xP3-eGFP} plasmid and in vitro-synthesized mRNA encoding the *piggyBac* transposase were pre-mixed at DNA/RNA concentrations of 500:300 ng/μl or 100:300 ng/μl and injected into the embryo center. Injections needles were pulled using borosilicate glass capillaries (Harvard Apparatus, 1.0 mm outer and 0.58 mm inner diameter). Needles were opened using forceps, backfilled, and injections were carried out as described above for *M. abdita*.

Inverse PCR

Genomic DNA was isolated from a pool of adult transgenic flies by SDS lysis and subsequent DNase-free RNase treatment (Andres and Thummel, 1994), digested with *Hae*III as described (Bellen et al., 2004), and self-circularized with T4 DNA Ligase. Fragments containing genomic DNA adjacent to the left *piggyBac* arm were amplified with primer pair PLF/PLR (Bellen et al., 2004), followed by a nested PCR with primer pair 5'-TTGTTGGTCAACTTCAAAGTCCAC/5'-CATGCGTCAATTTTACGCA-GACTA; fragments containing genomic DNA adjacent to the right *piggyBac* arm were amplified with primer pair PRF/PRR (Bellen et al., 2004), followed by a nested PCR with primer pair 5'-CATGATTATCTTTAACGTACGTCACAA/5'-TCAAAGTAGGAGCTTCTAAACGCT. The products of both nested PCRs were cloned into pCRII-TOPO (Invitrogen) and eight clones of each reaction were sequenced to determine the genomic locus 5' and 3' of the integrated *piggyBac* element.

Southern hybridization

DNA digestion, transfer, and probe hybridization were essentially performed following standard procedures (Sambrook and Russell, 2001). Briefly, genomic DNA was isolated from pools of the transgenic line and wild-type flies as described above, digested by *Xba*I over night, and separated on a 1% TAE agarose gel. The agarose gel was incubated for 20 min in denaturation buffer (0.5 N NaOH, 1.5 M NaCl), which was also used to transfer DNA to a positively charged nylon transfer membrane (GE Healthcare) over night. Following DNA transfer, the membrane was washed with neutralization buffer (0.5 M Tris Base, 1.5 M NaCl, pH 7.2-7.4), rinsed with 2x SSPE buffer (300 mM NaCl, 20 mM NaH_2PO_4-$1H_2O$, 2 mM Na_2EDTA, pH 7.4), and DNA was UV cross-linked to the membrane. A digoxigenin labeled probe against eGFP was synthesized with PCR DIG Probe Synthesis Kit (Roche) according to the manual. Following probe hybridization and incubation of alkaline phosphatase linked Fab fragments against digoxigenin (Roche, 1:20,000), CDP star (Bright Star Bio Detect, Ambion) was used for chemiluminescence-based immunodetection of alkaline phosphatase on a ChemoChem Imager (Intas).

Outcrossing

For three generations, out-crosses of transgenic against wild-type flies were set up in pools of either around ten wild-type virgin females with ten transgenic males or ten transgenic virgin females with ten wild-type males. In the third generation, embryos were analyzed for in vivo His2Av-mCherry expression at various embryonic stages.

Supplementary References

Amaya, E., Musci, T. J. and Kirschner, M. W. (1991). Expression of a dominant negative mutant of the FGF receptor disrupts mesoderm formation in Xenopus embryos. *Cell* **66**, 257–270.

Andres, A. J. and Thummel, C. S. (1994). Methods for quantitative analysis of transcription in larvae and prepupae. *Methods Cell Biol* **44**, 565–573.

Bellen, H. J., Levis, R. W., Liao, G., He, Y., Carlson, J. W., Tsang, G., Evans-Holm, M., Hiesinger, P. R., Schulze, K. L., Rubin, G. M., et al. (2004). The BDGP gene disruption project: single transposon insertions associated with 40% of Drosophila genes. *Genetics* **167**, 761–781.

Bire, S., Ley, D., Casteret, S., Mermod, N., Bigot, Y. and Rouleux-Bonnin, F. (2013). Optimization of the *piggyBac* transposon using mRNA and insulators: toward a more reliable gene delivery system. *PLoS ONE* **8**, e82559.

Handler, A. M. and Harrell, R. A. (1999). Germline transformation of *Drosophila melanogaster* with the *piggyBac* transposon vector. *Insect Mol Biol* **8**, 449–457.

Horn, C. and Wimmer, E. A. (2000). A versatile vector set for animal transgenesis. *Dev Genes Evol* **210**, 630–637.

Kapetanaki, M. G., Loukeris, T. G., Livadaras, I. and Savakis, C. (2002). High frequencies of *Minos* transposon mobilization are obtained in insects by using in vitro synthesized mRNA as a source of transposase. *Nucleic Acids Res* **30**, 3333–3340.

Kwan, K. M., Fujimoto, E., Grabher, C., Mangum, B. D., Hardy, M. E., Campbell, D. S., Parant, J. M., Yost, H. J., Kanki, J. P. and Chien, C.-B. (2007). The Tol2kit: a multisite gateway-based construction kit for Tol2 transposon transgenesis constructs. *Dev Dyn* **236**, 3088–3099.

Rafiqi, A. M., Lemke, S. and Schmidt-Ott, U. (2011a). *Megaselia abdita*: culturing and egg collection. *Cold Spring Harb Protoc* **2011**, pdb.prot5600.

Rafiqi, A. M., Lemke, S. and Schmidt-Ott, U. (2011b). *Megaselia abdita*: preparing embryos for injection. *Cold Spring Harb Protoc* **2011**, pdb.prot5601.

Sambrook, J. and Russell, D. W. (2001). *Molecular Cloning*. 3rd ed. Cold Spring Harbor Laboratory Press (New York).
